# Gait pattern in 9-11-year-old children with generalized joint hypermobility compared with controls; a cross-sectional study

**DOI:** 10.1186/1471-2474-14-341

**Published:** 2013-12-05

**Authors:** Helene Nikolajsen, Peter Kastmand Larsen, Erik Bruun Simonsen, Tine Alkjær, Simon Falkerslev, Jens Halkjær Kristensen, Bente Rona Jensen, Lars Remvig, Birgit Juul-Kristensen

**Affiliations:** 1 Department of Rheumatology (COHYPCO), University Hospital of Copenhagen, 2100, Copenhagen Ø, Denmark; 2 Department of Physiotherapy, University College South Denmark, Degnevej 16, 6705, Esbjerg Ø, Denmark; 3 Department of Forensic Medicine, Section of Forensic Pathology, University of Copenhagen, 2200, Copenhagen N, Denmark; 4 Department of Neuroscience and Pharmacology, University of Copenhagen, 2200, Copenhagen N, Denmark; 5 Department of Nutrition, Exercise and Sports, University of Copenhagen, 2200, Copenhagen N, Denmark; 6 Research Unit for Musculoskeletal Function and Physiotherapy, Institute of Sports Science and Clinical Biomechanics, University of Southern Denmark, Odense M, Denmark; 7 Institute of Occupational Therapy, Physiotherapy and Radiography, Department of Health Sciences, Bergen University College, Bergen, Norway

**Keywords:** Generalized joint hypermobility, Children, Gait analysis, Gait pattern, Kinetics, Kinematics

## Abstract

**Background:**

To study differences in gait patterns in 10-year-old children with Generalized Joint Hypermobility (GJH) and with no GJH (NGJH).

**Methods:**

A total of 37 children participated (19 GJH, 18 NGJH, mean age 10.2 (SD 0.5) years). Inclusion criteria for GJH were a Beighton score of ≥5, with at least one hypermobile knee joint; for NGJH a Beighton score of ≤4, and no hypermobile knees and for both groups no knee pain during the previous week. All children were recorded by five video cameras, while they walked across three force platforms. Net joint moments were calculated in 3D by inverse dynamics and peak values provided input to statistical analyses.

**Results:**

In the frontal plane, children with GJH had a significantly lower peak knee abductor moment and peak hip abductor moment. In the sagittal plane, the peak knee flexor moment and the peak hip extensor moment were significantly lower in the GJH group although the absolute difference was small.

**Conclusions:**

The walking pattern was the same for children with GJH and for healthy children, as there were no differences in kinematics, but it was, however, performed with different kinetics. Children with GJH walked with lower ankle, knee and hip joint moments compared to children with NGJH. However, the clinical importance of these differences during normal gait is unknown. To obtain this knowledge, children with GJH must be followed longitudinally.

**Trial registration:**

The study was approved by the Committee on Biomedical Research Ethics for Copenhagen and Frederiksberg, Denmark (jnr. KF01-2006-178).

## Background

Generalized Joint Hypermobility (GJH) has been described as a condition with ligamentous or capsular looseness, which is ‘inherent in a person’s make up and is determined by their fibrous protein genes’ [1]. Other studies have described GJH as a tissue adaptation due to repetitive movements to end range, as in e.g. ballet dancing [2]. However, there is still no consensus on the pathophysiology for GJH [3,4], although different genotypes are thought to contribute to some of the biological variation [5].

The Beighton Scoring system [6] is most often used to diagnose GJH [7]. It is reproducible [8] and it was found to be acceptable on concurrent validity against a goniometer in 6–12 year-old children [9]. Cut-points for classification of GJH according to The Beighton Scorings system vary for children, as both 4 [10], 5 [11], 6 [12], and 7 [9] positive tests out of 9 have been recommended. Two recent Danish cohort studies reported the prevalence of GJH among 7–8 and 10-year-old school children to be 29% and 36% (cut-point 4), 19% and 17% (cut-point 5) and 10% and 11% (cut-point 6) [13,14]. Discomfort due to GJH has previously been reported, most often among those diagnosed as Benign Joint Hypermobility Syndrome (BJHS) or other Hereditary Connective Tissue Disorders (HCTD) [15].

Several symptoms have been related to ‘hypermobility’ in children, such as arthralgia [16], growing pain [17], anterior knee pain syndrome, distortion, subluxation or dislocation episodes [18], soft tissue rheumatism [19], joint swelling often after physical activity [18], and severe cartilage damage in the knees [20]. Symptoms from the knee are described as the most frequent symptoms in relation to hypermobility [21,22]. In contrast, it has been stated that GJH might be an asset in certain elite sports [23]. However, it is still unclear why some children with GJH develop musculoskeletal symptoms, while others do not.

Several cross-sectional studies have indicated that children with GJH are clumsy in early childhood, characterized by delayed first walking and poor coordination [24], as well as reduced proprioception [25], reduced muscle strength [26] and reduced stamina [16]. Others could not confirm these motor deficits [13,14,27]. However, a longitudinal study has indicated that hypermobile children with lower limb pain at preadolescence (9–11 years) have an increased risk of pain recurrence 4 years later [28]. Several studies have indicated a relationship between GJH and osteoarthritis **(**OA) [7], but no longitudinal study has confirmed this relationship. Generally, factors contributing to the risk of OA are joint injuries [29], and GJH was reported to increase the risk of injury during contact sport [22].

A recent study found increased joint moments during walking in adults with GJH compared with adults without GJH (NGJH) [30], and adults with GJH had more pain, symptoms, trouble with daily activities, sports activities and decreased quality of life compared with healthy controls [31].

Since GJH is a hereditary condition and it is anticipated that symptoms develop over time, it is important to study whether or not there is an influence on GJH children at a relatively early stage. Such knowledge may be used to implement treatment strategies to reduce, delay the onset of, or prevent children with GJH from developing, symptoms such as pain, joint swelling or joint luxations [28].

Gait analysis is a widely used method to obtain objective data in 3D in the clinic in order to detect movement abnormalities in both children and adults. One study found a significantly altered gait pattern in children with BJHS compared with controls, i.e. increased knee extension in mid-stance [32]. Among children with BJHS, the most frequent symptom is pain, which is known to influence the gait pattern [33]. But children with GJH do not necessarily have pain, so whether this altered gait pattern holds true also for children with GJH is unknown. As previously described, increased joint moments during normal gait were found in adults with GJH [30]. It seems therefore relevant to investigate whether there is an influence on gait kinetics and kinematics in GJH children, since an altered gait pattern may constitute a risk factor for the development and/or progression of OA [34,35].

Thus, the purpose of the present study was to perform a clinical gait analysis on a group of children with GJH without knee pain compared with a group of healthy controls (NGJH). The hypothesis was that children with GJH walk with an altered gait pattern, probably with higher joint moments than children with NGJH.

## Methods

### Study design and tests

The study was cross-sectional, comprising children with GJH and without GJH (NGJH) in the 9–11 age range, corresponding to fourth grade in school. The children were recruited consecutively from the Copenhagen Hypermobility Cohort (COHYPCO) of school children [13,14].

### Subjects

Inclusion criteria for GJH were a Beighton score of ≥5, using a reproducible test performance [8] and at least one hypermobile knee. Inclusion criteria for NGJH were a Beighton score of ≤4, and for both groups, no knee pain within the previous week. Excluded from both groups were children with previous/current serious knee trauma, hereditary diseases like Ehlers-Danlos Syndrome, Marfan Syndrome, Osteogenesis Imperfecta, a body mass index (BMI) of >25, and an inability to understand Danish. All children had previously been examined clinically for GJH and BJHS [13,14] and at inclusion all children were clinically re-tested to verify the status of GJH or NGJH. In total, 39 children aged 9–11 were included, of which 19 were clinically classified as GJH (10 girls and 9 boys) and 20 as NGJH (9 girls and 11 boys). The parents of each participating child gave their informed written consent according to the Declaration of Helsinki before the tests were conducted, and each child gave oral consent to participation prior to the clinical examination. There was no risk of any harm, and the study was approved by the Committee on Biomedical Research Ethics for Copenhagen and Frederiksberg, Denmark (jnr. KF01-2006-178). There was no conflict of interest.

### Procedures

Anthropometric measurements were obtained and fifteen reflective markers were placed by the same researcher according to the marker set-up described by Vaughan et al. [36].

After a few instructions, the children walked barefoot on a 10 m long walkway with recessed force platforms. The subjects aimed for at gait velocity of 1.22 m/s ≈ 4.4 km/h, which was recorded by two sets of photocells and regulated by oral feedback. They were given unlimited rehearsal trials, and when the setting and the speed had been familiarized, recording took place. The three trials close to 4.4 km/h were included for data processing, and the gait velocity was thus kept constant in order to limit variation due to difference in velocity [37].

Five digital video cameras (Canon MV 600) operating at 50 frames per second were used to record the movements during walking. When the children passed a photocell placed in front of the force platform, an electronic audio-signal was conducted to the five video cameras to ensure synchronization of the cameras and the force platforms. Signals from the force platform were sampled at 1000 Hz, low-pass filtered at 50 Hz and later reduced to 50 Hz to match the video recordings.

The children walked freely, and trials in which the children stepped with their entire foot on the first force platform (AMTI OR6-5-1, size 51, 0 × 46, 5 cm^2^) were used for calculation. All calculations were limited to the first stance phase of the right leg.

The Ariel Performance Analysis System (APAS, Ariel Dynamics Inc., San Diego, CA, USA) was used for digitization, and three-dimensional marker coordinates were reconstructed by direct linear transformation.

A Butterworth low-pass filter (fourth-order) with a cut-off frequency of 6 Hz was used on the movement data. Video analyses and calculations of kinematics and kinetics were performed without reference to health status.

Net joint moments were calculated by inverse dynamics according to Vaughan et al. [36]. Further parameters were anatomical joint angles, peak ground reaction forces, step length (from right heel marker (calcaneus) at heel strike to the left heel markers (calcaneus) at heel strike), step width (the max distance between the two lateral malleolus) and foot progression angle. The latter was defined as the angle between the line of walking progression and a line between the lateral malleolus and the fifth metatarsal joint.

All calculations were limited to one stance phase of the right leg during gait per trial. (All children had bilateral hypermobile knees). The children walked along the walkway several times and three successful trials per child were selected for further analysis. A successful trial was defined by correct heel strike on the force platform and correct walking speed (1.22 m/s ±5%). Data from two children had to be excluded because of technical problems with the data sampling procedures. Thus, data from 37 children, 18 NGJH (8 girls and 10 boys) and 19 GJH (10 girls and 9 boys) were subjected to further analysis (Table 1).

**Table 1:** Demographic data

	**NGJH (n = 18)**	**GJH (n = 19)**	**p-value**
**Boys/girls**	10/8	9/10	
**Beighton Score** (0–9 points)	1.1 (1.1)	7.6 (1.0)	**<0.001**
**Age** (years)	10.2 (0.4)	10.1 (0.5)	0.66
**Body height** (m)	1.47 (0.05)	1.45 (0.06)	0.15
**Body mass** (kg)	36.2 (5.7)	36.4 (8.5)	0.92
**BMI**	16.6 (2.3)	17.2 (2.8)	0.53
**Hours/week with sport**	2.6 (2.3)	3.5 (2.3)	0.25

Mean (SD) for children with Non-Generalized Joint Hypermobility (NGJH) and Generalized Joint Hypermobility (GJH). Significant differences (p-values <0.05) are marked in bold.

Moments were normalized as follows: peak value (Nm)/(body mass (kg) * height (m)) * 100.

Ground reaction forces were calculated in three directions and normalized to % of body weight as follows: peak value (N)/(body mass (kg) * 9.81) * 100.

### Questionnaire

All subjects filled out a questionnaire regarding weekly physical activity (yes/no) and number of weekly hours with physical activity.

### Statistics

Data were tested to be normally distributed by the Kolmogorov–Smirnov test. Differences in demographic values were tested by an independent-sample t-test for continuous variables (height, weight, weekly hours of sports), and for categorical variables (participation in sports, yes/no) a Chi^2^ test was used.

Moments, angles and ground reaction forces (GRF) were analyzed by linear Mixed Model regression analysis for main effects, dependent variables (one at a time) were moments, angles and GRF; fixed factors were status (GJH/NGJH), gender (M/F) and sports participation (yes/no). Body mass index (BMI) was a covariate. The subjects’ ID-numbers and trial numbers were treated as random and repeated factors, respectively. The level of significance was p < 0.05, and all statistical analyses were performed in SPSS (Statistical Package for Social Sciences, Inc. Chicago, IL, USA, version 19.0).

## Results

The Beighton score was 7.6 (SD 1.0) and 1.1 (SD 1.1) for GJH and NGJH, respectively. There were no significant differences in demographic variables (age, height, weight and BMI) between groups (Table 1). The average walking speed for all the children was 1.22 m/s (SD 0.04) with no significant difference between the two groups. Likewise, no significant difference was observed regarding step length and foot progression angle, but GJH walked with a smaller step width (p < 0.001) of only 8 mm, which is most likely of no clinical relevance.

**Figure 1:**
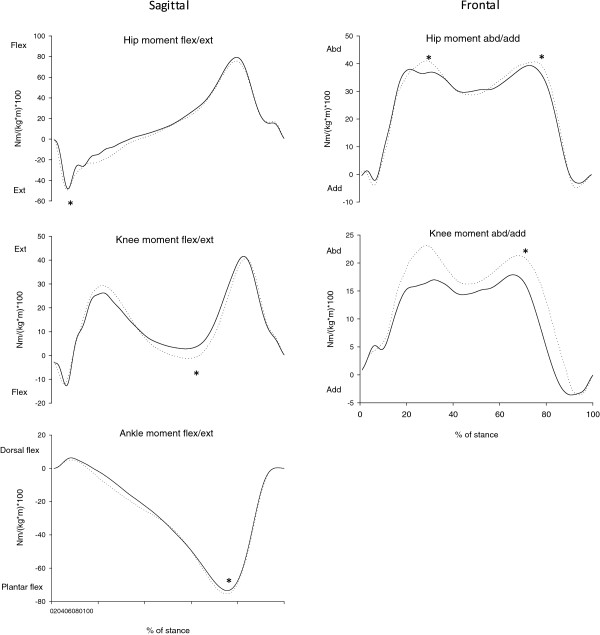
**Joint moments.** Mean internal joint moments for the two groups of children, in the sagittal and frontal plane for right/hypermobile leg, i.e. hip, knee and ankle during stance. Moments are normalized to body mass and body height **(**Nm/(kg*m)*100) and the duration of the stance phase is normalized to 100%. Solid lines are children with generalized joint hypermobility (GJH) and dotted lines represent the healthy control children (NGJH). Asterisks indicate significant between-group differences (p-values <0.05).

Several differences in peak joint moments were found (Figure 1). In the frontal plane, children with GJH had a lower second peak knee abductor moment than NGJH, 23.1 (SD 8.3) Nm/(kg*m)*100 vs. 29.2 (SD 9.3) Nm/(kg*m)*100 (p = 0.003) (Table 2) and a lower first and second peak hip abductor moment of 46.5 (SD 6.9) Nm/(kg*m)*100 vs. 48.4 (SD 8.7) Nm/(kg*m)*100 (p = 0.007) and 45.2 (SD 8.3) Nm/(kg*m)*100 vs. 48.5 (SD 10.6) Nm/(kg*m)*100 (p = 0.017), respectively. In the sagittal plane, children with GJH had a lower third peak knee flexor moment during mid stance, -0.8 (SD 8.9) Nm/(kg*m)*100 vs. -6.7 (SD 13.1) Nm/(kg*m)*100 (p = 0.005), as well as a lower first peak hip extensor moment, -57.8 (SD 10.7) Nm/(kg*m)*100 vs. -64.7 (SD 14.6) Nm/(kg*m)*100 (p = 0.006). Children with GJH had a lower ankle plantar flexor moment, -82.9 (SD 14.1) Nm/(kg*m)*100 vs. -83.4 (SD 8.3) Nm/(kg*m)*100 (p = 0.029), but this is most likely clinically insignificant.

**Table 2:** Step width and length, foot progression angle (FPA) and peak joint moments in sagittal and frontal plane.

	**NGJH n = 18**	**GJH n = 19**	**p-value**
	**Mean (SD)**	**Mean (SD)**	
**Step width (max)** (m)	0.13 (0.01)	0.12 (0.01)	**p < 0.001**
**Step length** (m)	0.61 (0.02)	0.62 (0.04)	p = 0.886
**Right FPA** (degrees)	8.6 (4.1)	8.1 (5.9)	p = 0.531
**Left FPA** (degrees)	−9.7 (5.6)	−8.4 (6.8)	p = 0.652
**Peak joint moments:**			
***Sagittal plane***			
**Ankle (sA1) (**Nm/(kg*m)*100)	7.3 (3.0)	9.4 (5.3)	p = 0.182
**Ankle (sA2) (**Nm/(kg*m)*100)	−83.4 (8.3)	−82.9 (14.1)	**p = 0.029**
**Knee (sK1) (**Nm/(kg*m)*100)	−19.6 (7.9)	−17.2 (8.0)	p = 0.050
**Knee (sK2) (**Nm/(kg*m)*100)	30.4 (11.8)	30.4 (13.6)	p = 0.781
**Knee (sK3) (**Nm/(kg*m)*100)	−6.7 (13.1)	−0.8 (8.9)	**p = 0.005**
**Knee (sK4) (**Nm/(kg*m)*100)	44.3 (11.1)	44.8 (15.6)	p = 0.851
**Hip (sH1) (**Nm/(kg*m)*100)	−64.7 (14.6)	−57.8 (10.7)	**p = 0.006**
**Hip (sH2) (**Nm/(kg*m)*100)	86.3 (15.7)	92.8 (21.8)	p = 0.460
***Frontal plane***			
**Knee (fK1) (**Nm/(kg*m)*100)	28.2 (9.4)	25.1 (8.8)	p = 0.073
**Knee (fK2) (**Nm/(kg*m)*100)	29.2 (9.3)	23.1 (8.3)	**p = 0.003**
**Hip (fH1) (**Nm/(kg*m)*100)	48.4 (8.7)	46.5 (6.9)	**p = 0.007**
**Hip (fH2) (**Nm/(kg*m)*100)	48.5 (10.6)	45.2 (8.3)	**p = 0.017**

Mean (SD) of step length, width, and foot progression angle (FPA) are shown. Further, mean (SD) of normalized **(**Nm/(kg*m)*100) internal peak joint moments for right/hypermobile leg during stance phase, for NGJH (Non-Generalized Joint Hypermobility) and GJH (Generalized Joint Hypermobility) children, with p-values for effect of status. In sagittal plane positive/negative values designate dorsal-/plantar flexion in the ankle, extension/flexion in the knee and flexion/extension in the hip. In frontal plane positive/negative values designate abduction/adduction for both knee and hip. Significant differences (p-values <0.05) are marked in bold.

s = sagittal plane, f = frontal plane; A = ankle, K = knee, H = hip; 1,2,3,4 = peak number

The two peaks on the vertical ground reaction force were significantly lower in children with GJH (p = 0.002; p = 0.025, Table 3), but the differences are small.

**Table 3:** Peak ground reaction forces

	**NGJH n = 18**	**GJH n = 19**	**p-value**
	**Mean (SD)**	**Mean (SD)**	
***Vertical***			
**Fz1** (% BW)	112.3 (8.0)	109.8 (9.3)	**p = 0.002**
**Fz2** (% BW)	80.3 (6.3)	81.1 (4.4)	p = 0.079
**Fz3** (% BW)	116.3 (7.3)	115.7 (8.7)	**p = 0.025**
***Ant-post***			
**Fy1** (% BW)	−14.6 (2.9)	−14.4 (3.2)	p = 0.707
**Fy2** (% BW)	21.3 (2.2)	21.7 (2.4)	p = 0.642
***Med-lat***			
**Fx1 (max)** (% BW)	3.4 (1.4)	3.6 (2.1)	p = 0.767
**Fx1 (min)** (% BW)	- 4.1 (1.3)	- 4.3 (1.2)	p = 0.579

Mean (SD) peak ground reaction forces for right/hypermobile leg during stance phase normalized to % Body Weight N/(kg*9.81) *100, for NGJH (Non-Generalized Joint Hypermobility) and GJH (Generalized Joint Hypermobility) children, with p-values for effect of status. Significant differences (p-values <0.05) are marked in bold.

F = Force z = vertical direction, y = anterior-posterior direction, x = medial-lateral direction 1,2,3 = peak number.

**Figure 2:**
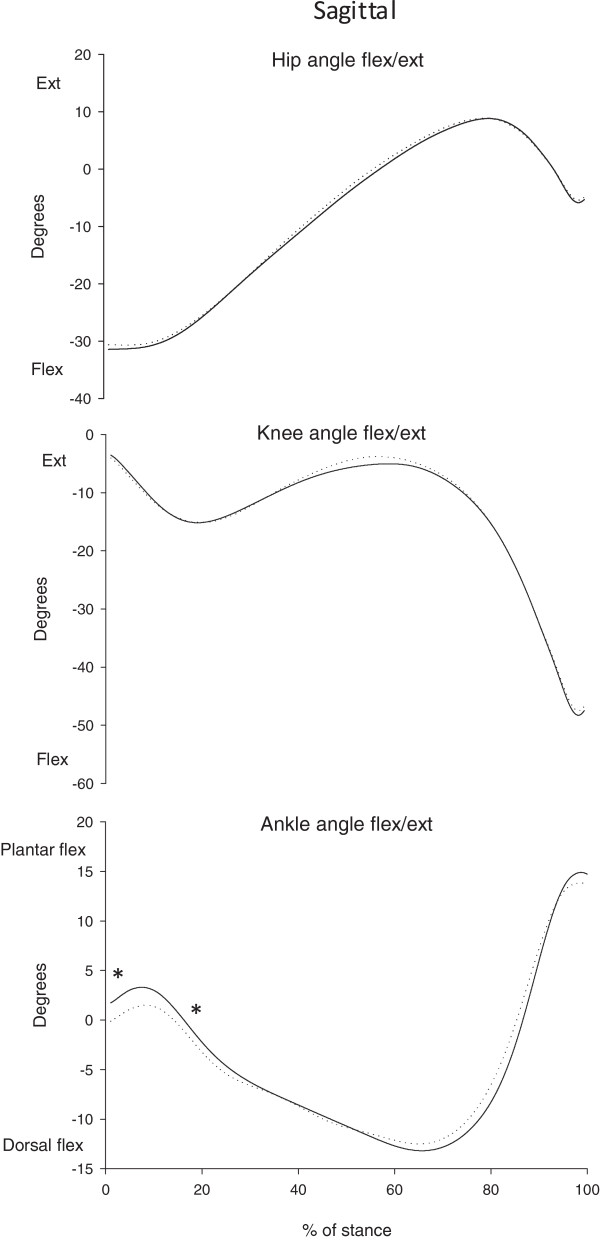
**Joint angles.** Mean joint angles for the two groups of children, in the sagittal plane and for right/hypermobile leg, i.e. hip, knee and ankle during stance. The duration of the stance phase is normalized to 100%. Solid lines are children with generalized joint hypermobility (GJH) and dotted lines represent the healthy control children (NGJH). Asterisks indicate significant between-group differences (p-values <0.05).

Regarding joint angles (Figure 2), there were no differences in the knee and hip angles between the two groups, but at the ankle joint the GJH group was significantly more plantar flexed at heel strike (1.5° (SD 7.2) vs. -0.1° (SD 4.9), p = 0.034) and less plantar flexed at peak knee flexion angle (1.9° (SD 6.2) vs. 3.4° (SD 5.8), p = 0.048) (Table 4). The differences in ankle angles is less than 2 degrees, which is most likely of no clinical relevance.

**Table 4:** Peak joint angles during stance

	**NGJH n = 18**	**GJH n = 19**	**p-value**
	**Mean (SD)**	**Mean (SD)**	
***Sagittal plane***			
**Mean hip** (degrees)	−8.7 (8.1)	−9.1 (6.9)	p = 0.445
**Mean knee** (degrees)	−14.1 (6.7)	−14.3 (5.9)	p = 0.943
**Mean ankle** (degrees)	−4.2 (4.9)	−4.2 (5.7)	p = 0.498
**ROM hip** (degrees)	41.6 (5.4)	41.7 (5.5)	p = 0.329
**ROM knee** (degrees)	51.5 (5.8)	51.5 (5.5)	p = 0.859
**ROM ankle** (degrees)	29.6 (6.8)	31.0 (5.9)	p = 0.454
**Hip angle at heel strike** (degrees)	−30.6 (8.4)	−31.5 (6.7)	p = 0.262
**Knee angle at heel strike** (degrees)	−4.0 (6.8)	−3.7 (5.5)	p = 0.580
**Ankle angle at heel strike** (degrees)	−0.1 (4.9)	1.5 (7.2)	**p = 0.034**
**Peak knee flexion angle** (degrees)	−15.6 (9.1)	−15.4 (6.7)	p = 0.915
**Hip angle at peak knee flex** (degrees)	−26.2 (9.9)	−26.8 (5.9)	p = 0.186
**Ankle angle at peak knee flex** (degrees)	3.4 (5.8)	1.9 (6.2)	**p = 0.048**
**Peak knee extension** (degrees)	−2.5 (7.6)	−3.8 (6.4)	p = 0.663

Mean (SD) of peak joint angles (in degrees) for right/hypermobile leg during stance phase, for NGJH (Non-Generalized Joint Hypermobility) and GJH (Generalized Joint Hypermobility) children, with p-values for effect of status. Zero refers to normal anatomical position. Positive/negative values designate plantar-/dorsal flexion in the ankle, extension/flexion for both knee and hip. Significant differences (p-values <0.05) are marked in bold.

## Discussion

The walking pattern was the same for children with GJH and for healthy children, as there were no clinically relevant differences in kinematics, but walking was, however, performed with different kinetics, represented by lower peak joint moments for knee and hip joint.

The hypothesis that children with GJH had elevated joint moments could not be confirmed. In contrast, children with GJH presented decreased joint moments compared with NGJH children.

Although all subjects walked at the same speed, differences in the joint moments were observed. This is in agreement with previous studies of human walking [38]. Differences in co-contractions around the joint could also be an explanation. Net joint moments are highly influenced by the degree of co-contraction [39], but unfortunately electromyography **(**EMG) was not measured in the present study. However, as a parallel to the present study, the same children with GJH showed increased co-contraction in the muscles around the knee joint (m. rectus femoris and hamstrings) during an isometric submaximal sitting knee flexion task [40].

A recent study supports the statement that subjects with GJH have a need for increased co-contraction around the knee joint compared with controls [41]. Adults with pain-free BJHS had significantly higher levels of co-contraction over the knee than subjects with normal flexibility, due to an increased semitendinosus activity during standing [41]. The present study comprised a dynamic walking task, which may even imply further demands for knee joint stabilization and possibly co-contraction.

Generally, subjects who are younger, inexperienced or with a low physical activity level present increased co-contraction levels, possibly as a potential motor control strategy to maintain dynamic knee stability and protect against excessive joint loads [42]. Joint stability through co-contractions may be necessary when the joint experiences high distraction or shear forces and/or when the passive structures are compromised. The passive structures in subjects with GJH may be compromised or more extensible than in subjects without GJH. Thus, increased co-contraction or an altered muscle strategy may be a motor control strategy to prevent instability.

The suggested relationship between GJH and OA is still unclear, but studies of other patient groups indicate that the knee abductor moment is of relevance in the development OA. Several studies of subjects with knee problems (ACL-reconstruction, trans-tibial amputation) have shown increased peak abduction moments in the ACL-affected and the non-prosthetic knee, that may predispose for premature joint degeneration and early onset of medial knee OA [43,44]. This is in agreement with an earlier study, which reported the risk of progression of knee OA to be 6.46 times higher with a 1% increase in the internal knee abductor moment [45]. The study also showed that baseline knee abductor moment, reflecting the dynamic load on the medial compartment, could predict radiographic medial OA progression at a six-year follow-up in patients with medial compartment knee OA [45].

However, in the present study, a 21% lower knee abductor moment was found in children with GJH and the long-term consequence of this altered gait pattern is unknown. One may speculate whether or not a decreased knee abductor moment is a risk factor for lateral knee OA, as suggested in a study of patients with lateral knee OA who presented 63% lower knee abductor moments compared with healthy controls [46]. As the present study was cross-sectional, it is not possible to conclude that the decreased knee abductor moment could be a predictor for lateral knee OA. Future research should be directed towards longitudinal studies to explain causal mechanisms.

The present study showed no group differences in hip and knee angles during normal gait, where especially the knee angles in the sagittal plane were expected to differ between groups. This means that even though the current GJH children had bilateral hypermobile knee joint this did not seem to influence the knee joint angle during normal walking. This is in contrast to the only comparable study, which showed a lower peak knee flexion angle in the first half of the stance phase, and an increased knee extension in mid stance during walking in 8–15 year-old children with BJHS compared with controls [32]. A possible explanation for this could be the different study populations, since the children with BJHS presented multiple joint pains, as opposed to the current pain free children with GJH.

Measurement errors on reflective markers are reported to be a few mm [47,48]. Inaccurracies in calculated parameters are unknown. The current motion capture system seemed to be highly accurate compared to other similar systems [47]. Generally, the repeatability is known to be more reliable in kinetic data than kinematic data [49]. Kadaba et al. performed a test-retest study on joint moments in 3D and found that the moments in the lower extremities are highly reproducible [50]. A systematic review concluded that for clinical use at least two degrees margin of error must be accepted [48], therefore clinical relevance of the small difference in ankle joint position is assumed to be low. However, the present data are in line with a recent study which reported that EDS-patients of hypermobile type (EDS-HT) also had both increased plantar flexion angle and plantar flexor moment during the stance phase of walking compared with healthy controls [51]. Thus, both the study of adults with EDS-HT and the present study of children with GJH may indicate a general limited push-off ability of the ankle for subjects with GJH.

Although the two peaks on the vertical ground reaction force were significant, we do not consider this as clinically relevant as the relative differences are at a level of only 2.5% and 0.5%. Studies with small between-groups differences (i.e. %) is a common point of discussion using mixed models; as statistics are performed on all 3 trials (repeated factor) it can lead to statistical significance.

However, a direct explanation of the small differences between statistics and figures is that three trials for each subject provided input to the Mixed Model, while the mean values presented under results and in the figures are averaged over three trials for each subject and across subjects. However, it is considered an advantage to use the Mixed Model approach as more information is provided for the statistical test.

The strength of the present study is the very strict inclusion and exclusion criteria to ensure group homogeneity. All tests were performed according to prescribed standardized procedures and furthermore, the walking speed was standardized to be the same across all trials. Incorrect marker set-up is thought to imply a large bias in 3D gait analysis [48], therefore all anthropometric measurements and marker set-ups were performed by the same researcher, to reduce this risk of bias. Further, all researchers were blinded to the status of all subjects during testing and analyzing data.

## Conclusion

To our knowledge this study is the first to examine the gait pattern in pain-free children with GJH.

The walking pattern was the same for children with GJH and for healthy children, as there were no clinically relevant differences in kinematics, but it was however performed with different kinetics. Children with GJH showed lower knee and hip peak joint moments in sagittal and frontal plane than children with NGJH during normal gait. The clinical importance of this altered walking pattern is unknown, and to obtain this knowledge children with GJH must be followed longitudinally to see if they develop e.g. pain or OA.

## Abbreviations

GJH: Generalized joint hypermobility; NGJH: No generalized joint hypermobility; BJHS: Benign joint hypermobility syndrome; HCTD: Hereditary connective tissue disorders; OA: Osteoarthritis; GRF: Ground reaction force; BMI: Body mass index; EMG: Electromyography; ACL: Anterior cruciate ligament.

## Competing interests

The authors declare that they have no competing interests.

## Authors’ contributions

BJK, LR, JHK, EBS, TA and BRJ contributed to the conception and design of the study. BJK provided funding and approvals. HN, BJK, SF and EBS performed the gait analysis of the children. HN, PKL, TA, and BJK performed the statistical analyses. HN drafted the manuscript and all authors critically revised the manuscript and approved the final version.

## Pre-publication history

The pre-publication history for this paper can be accessed here:

http://www.biomedcentral.com/1471-2474/14/341/prepub
